# *N*,*N*′-[2,2′-(Phenyl­aza­nedi­yl)bis­(ethane-2,1-di­yl)]dipicolinamide

**DOI:** 10.1107/S1600536813008696

**Published:** 2013-04-10

**Authors:** Gao-Nan Li, Zhi-Gang Niu, Mei-Qi Huang, Ying Zou, Liang-Jiang Hu

**Affiliations:** a College of Chemistry and Chemical Engineering, Hainan Normal University, Haikou 571158, People’s Republic of China

## Abstract

The asymmetric unit of the title compound, C_22_H_23_N_5_O_2_, contains two independent mol­ecules with similar conformations; the terminal pyridine rings are oriented at dihedral angles of 23.99 (8) and 18.07 (8)° with respect to the central benzene ring in one mol­ecule and 28.99 (8) and 23.22 (8)° in the other. In the crystal, N—H⋯O and weak C—H⋯O hydrogen bonds link the mol­ecules into a three-dimensional supra­molecular structure. Weak inter­molecular C—H⋯π inter­actions are also observed in the crystal.

## Related literature

For background to bis­(pyridine­carboxamide) derivatives, see: Cornman *et al.* (1999 ▶); Song *et al.* (2010 ▶); Singh *et al.* (2008 ▶). For the synthesis, see: Jain *et al.* (2004 ▶); Lee *et al.* (2006 ▶); Barnes *et al.* (1978 ▶). For related structures, see: Adolph *et al.* (2012 ▶); Munro & Wilson (2010 ▶); Yan *et al.* (2012 ▶).
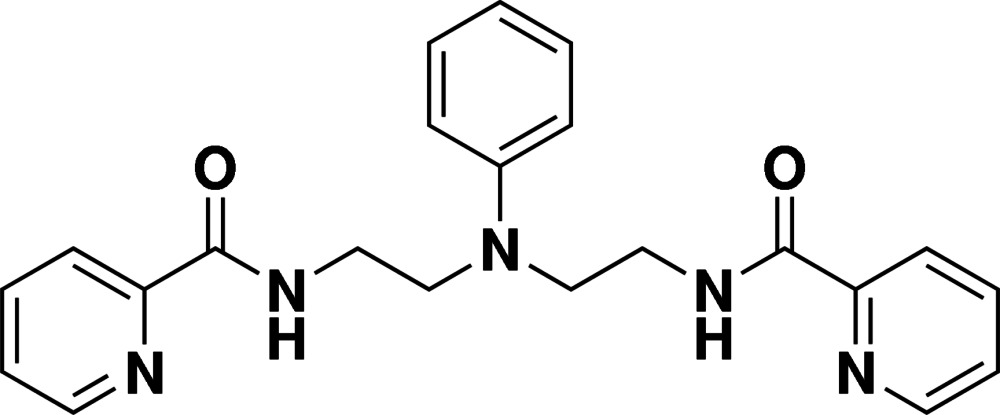


## Experimental

### 

#### Crystal data

C_22_H_23_N_5_O_2_
*M* *_r_* = 389.45
Monoclinic, 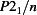

*a* = 8.64349 (7) Å
*b* = 24.8210 (3) Å
*c* = 18.40861 (18) Å
β = 90.5648 (8)°
*V* = 3949.20 (7) Å^3^
*Z* = 8
Cu *K*α radiation
μ = 0.70 mm^−1^
*T* = 100 K
0.12 × 0.08 × 0.07 mm

#### Data collection

Agilent Xcalibur Atlas Gemini ultra diffractometer
Absorption correction: multi-scan (*CrysAlis PRO*; Agilent, 2012 ▶) *T*_min_ = 0.886, *T*_max_ = 0.950
28468 measured reflections
7050 independent reflections
6128 reflections with *I* > 2σ(*I*)
*R*_int_ = 0.038

#### Refinement

*R*[*F* ^2^ > 2σ(*F* ^2^)] = 0.044
*wR*(*F* ^2^) = 0.097
*S* = 1.14
7050 reflections
523 parameters
H-atom parameters constrained
Δρ_max_ = 0.17 e Å^−3^
Δρ_min_ = −0.26 e Å^−3^

### 

Data collection: *CrysAlis PRO* (Agilent, 2012 ▶); cell refinement: *CrysAlis PRO*; data reduction: *CrysAlis PRO*; program(s) used to solve structure: *SHELXS97* (Sheldrick, 2008 ▶); program(s) used to refine structure: *SHELXL97* (Sheldrick, 2008 ▶); molecular graphics: *DIAMOND* (Brandenburg, 1999 ▶); software used to prepare material for publication: *OLEX2* (Dolomanov *et al.*, 2009 ▶).

## Supplementary Material

Crystal structure: contains datablock(s) global, I. DOI: 10.1107/S1600536813008696/xu5691sup1.cif

Structure factors: contains datablock(s) I. DOI: 10.1107/S1600536813008696/xu5691Isup2.hkl

Additional supplementary materials:  crystallographic information; 3D view; checkCIF report

## Figures and Tables

**Table 1:** Hydrogen-bond geometry (Å, °) *Cg*5 and *Cg*6 are the centroids of the C9–C14 benzene and C31–C36 benzene rings, respectively.

*D*—H⋯*A*	*D*—H	H⋯*A*	*D*⋯*A*	*D*—H⋯*A*
N2—H2⋯O3	0.86	2.44	3.1099 (18)	135
N7—H7⋯O1^i^	0.86	2.42	3.0998 (18)	136
C24—H24⋯O2^ii^	0.93	2.48	3.311 (2)	149
C25—H25⋯O4^iii^	0.93	2.54	3.213 (2)	129
C8—H8*A*⋯*Cg*6^iv^	0.97	2.72	3.6468 (18)	161
C30—H30*B*⋯*Cg*5^v^	0.97	2.74	3.6584 (18)	159

Symmetry codes: (i) 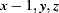; (ii) 
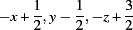
; (iii) 
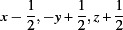
; (iv) 
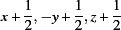
; (v) 
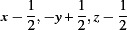
.

**Fig. 1.**
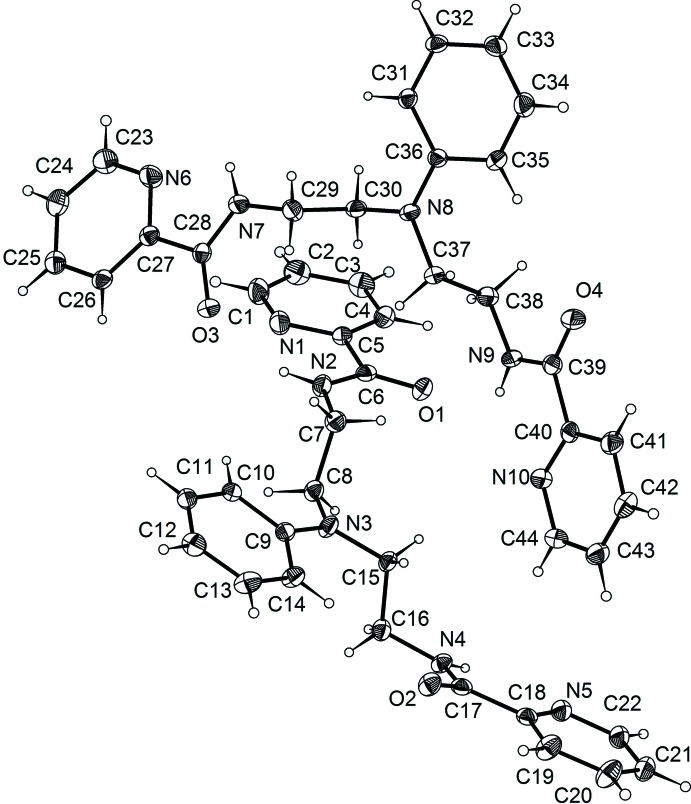
The molecular structure of (I), showing the atom-labelling scheme. Displacement ellipsoids are drawn at the 50% probability level and H atoms are shown as small spheres of arbitrary radii.

**Fig. 2.**
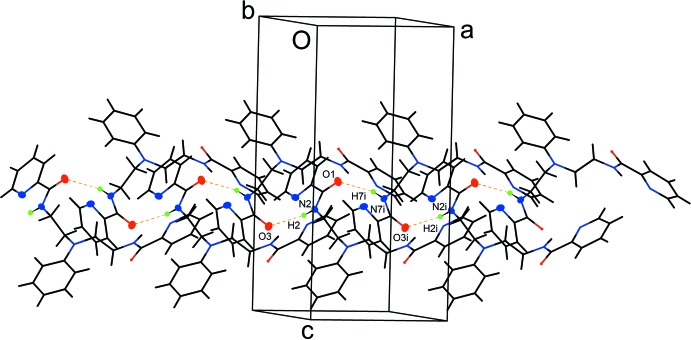
Representation of part of the lattice contents of (I), viewed approximately down the cell diagonal bisecting the b O a angle. Only selected interactions are shown for clarity. Atoms involved in hydrogen bonds are shown as balls of arbitrary radii. All other atoms and covalent bonds are represented as wires or sticks. [symmetry code: (i) 1+ x, y, z].

**Fig. 3.**
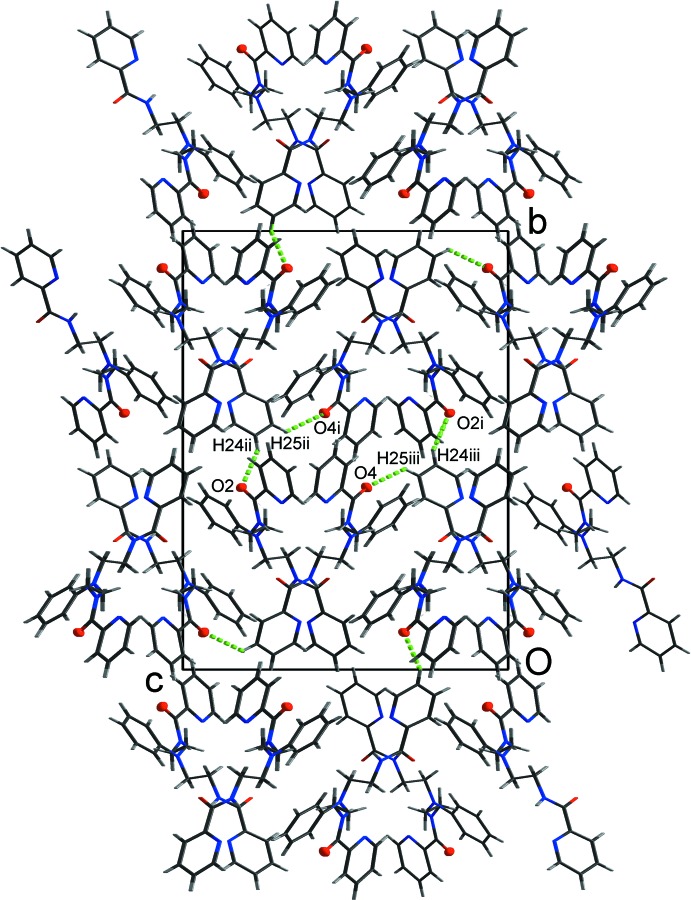
Representation of part of the lattice contents of (I), along the a axis, showing the hydrogen-bonded chains. Only selected interactions are shown for clarity. Atoms involved in hydrogen bonds are shown as balls of arbitrary radii. All other atoms and covalent bonds are represented as wires or sticks. [symmetry code: (i) 1–x, 1–y, 1–z; (ii) 1/2–x, 1/2+y, 3/2–z; (iii) 1/2+x, 1/2–y, –1/2+z].

## supplementary crystallographic information

### Comment

Bis(2-pyridinecarboxamide) derivatives comprise a large group of organic compounds that are ideal for the mono- or dinuclear chelation of metal ions (Cornman *et al.*, 1999; Song *et al.*, 2010; Singh *et al.*, 2008). In order to explore coordination chemistry of bis(pyridinecarboxamide) ligands, we have synthesized the title compound (I) and report here its crystal structure.

The asymmetric unit of the title compound (I), C_22_H_23_N_5_O_2_, contains two independent molecules with the similar structure, two terminal pyridine rings are oriented with respect to the central benzene ring at 23.99 (8) and 18.07 (8)° in one molecule and at 28.99 (8) and 23.22 (8)° in the other (Fig. 1). Every independent molecule contains two symmetrical N-ethyl(pyridine-2-carboxiamide) moieties linked by a phenylamino bridge. It is noteworthy that the C═O bonds are oriented trans to the pyridine nitrogen atom, which are in accord with those reported for previous structures (Munro *et al.*, 2010; Yan *et al.*, 2012). In the crystal, intermolecular N–H···O (Fig. 2) and weak C—H···O hydrogen bonds (Fig. 3) link the molecules into the three-dimensional supramolecular structure. The crystal packing exhibits also weak intermolecular C–H···π interaction, proved by short distance C30–H30B···Cg5 [3.6584 (18) Å] and C8–H8A···Cg6 [3.6468 (18) Å], where Cg5 and Cg6 are the centroids of the C9–benzene and C31–benzene rings, respectively [symmetry code: (iv) x+1/2, –y+1/2, z+1/2; (v) x–1/2, –y+1/2, z–1/2] (Table 1).

### Experimental

*N*-(3-Dimethylaminopropyl)-*N*-ethylcarbodiimide hydrochloride (EDCI, 375 mg, 1.95 mmol) and hydroxybenzotriazole (HOBt, 440 mg, 3.26 mmol) were added to a solution of picolinic acid (200 mg, 1.62 mmol) in dry DMF (10 ml) at room temperature. After the mixture was stirred for 30 min, *N*^1^-(2-aminoethyl)-*N*^1^-phenylethane-1,2-diamine (133 mg, 0.74 mmol) was added (Lee *et al.*, 2006). The reaction mixture was stirred overnight under N_2_ atmosphere. H_2_O (30 ml) was added to quench the reaction, and the mixture was extracted with ethyl acetate (3×20 ml). The combined organic phase was washed with brine, dried over Na_2_SO_4_, filtrated and concentrated in vacuum. The residue was purified by column chromatography (PE:EA = 2:1~1:2) to give compound (I) (264 mg, yield: 91.8%) as a white solid. Colorless single crystals suitable for X-ray structural analysis were obtained by slow evaporation of a mixture solution of dichloromethane and methanol at room temperature. ^1^H NMR (400 MHz, CDCl_3_): 3.48~3.61 (m, 8H), 6.62 (t, *J* = 8.0 Hz, 1H), 6.85 (d, *J* = 7.2 Hz, 2H), 7.15 (t, *J* = 8.0 Hz, 2H), 7.28~7.32 (m, 2H), 7.73 (t, *J* = 8.0 Hz, 2H), 8.09 (d, *J* = 7.2 Hz, 2H), 8.30~8.40 (m, 4H).

### Refinement

H-atoms were placed in calculated positions (C–H 0.93–0.97 Å, N–H 0.86 Å), and were included in the refinement in the riding model, with *U*_iso_(H) = 1.2*U*_eq_(C,N).

### Crystal data

**Table:**

C_22_H_23_N_5_O_2_	*F*(000) = 1648
*M**_r_* = 389.45	*D*_x_ = 1.310 Mg m^−^^3^
Monoclinic, *P*2_1_/*n*	Cu *K*α radiation, λ = 1.5418 Å
Hall symbol: -P 2yn	Cell parameters from 13020 reflections
*a* = 8.64349 (7) Å	θ = 3.0–67.1°
*b* = 24.8210 (3) Å	µ = 0.70 mm^−^^1^
*c* = 18.40861 (18) Å	*T* = 100 K
β = 90.5648 (8)°	Block, colorless
*V* = 3949.20 (7) Å^3^	0.12 × 0.08 × 0.07 mm
*Z* = 8	

### Data collection

**Table:**

Agilent Xcalibur Atlas Gemini ultra diffractometer	7050 independent reflections
Radiation source: Enhance Ultra (Cu) X-ray Source	6128 reflections with *I* > 2σ(*I*)
Mirror monochromator	*R*_int_ = 0.038
Detector resolution: 10.5095 pixels mm^-1^	θ_max_ = 67.2°, θ_min_ = 3.0°
ω scans	*h* = −8→10
Absorption correction: multi-scan (*CrysAlis PRO*; Agilent, 2012)	*k* = −29→29
*T*_min_ = 0.886, *T*_max_ = 0.950	*l* = −21→21
28468 measured reflections	

### Refinement

**Table:**

Refinement on *F*^2^	Primary atom site location: structure-invariant direct methods
Least-squares matrix: full	Secondary atom site location: difference Fourier map
*R*[*F*^2^ > 2σ(*F*^2^)] = 0.044	Hydrogen site location: inferred from neighbouring sites
*wR*(*F*^2^) = 0.097	H-atom parameters constrained
*S* = 1.14	*w* = 1/[σ^2^(*F*_o_^2^) + (0.0328*P*)^2^ + 1.7734*P*] where *P* = (*F*_o_^2^ + 2*F*_c_^2^)/3
7050 reflections	(Δ/σ)_max_ < 0.001
523 parameters	Δρ_max_ = 0.17 e Å^−^^3^
0 restraints	Δρ_min_ = −0.26 e Å^−^^3^

### Special details

**Table:**

Geometry. All e.s.d.'s (except the e.s.d. in the dihedral angle between two l.s. planes) are estimated using the full covariance matrix. The cell e.s.d.'s are taken into account individually in the estimation of e.s.d.'s in distances, angles and torsion angles; correlations between e.s.d.'s in cell parameters are only used when they are defined by crystal symmetry. An approximate (isotropic) treatment of cell e.s.d.'s is used for estimating e.s.d.'s involving l.s. planes.
Refinement. Refinement of *F*^2^ against ALL reflections. The weighted *R*-factor *wR* and goodness of fit *S* are based on *F*^2^, conventional *R*-factors *R* are based on *F*, with *F* set to zero for negative *F*^2^. The threshold expression of *F*^2^ > σ(*F*^2^) is used only for calculating *R*-factors(gt) *etc*. and is not relevant to the choice of reflections for refinement. *R*-factors based on *F*^2^ are statistically about twice as large as those based on *F*, and *R*- factors based on ALL data will be even larger.

### Fractional atomic coordinates and isotropic or equivalent isotropic displacement parameters (Å^2^)

**Table:**

	*x*	*y*	*z*	*U*_iso_*/*U*_eq_	
O3	0.01667 (13)	0.20717 (5)	0.70802 (6)	0.0213 (3)	
O4	0.42663 (14)	0.41541 (5)	0.43618 (7)	0.0262 (3)	
N10	0.72634 (16)	0.38111 (6)	0.56121 (7)	0.0211 (3)	
N8	0.05731 (16)	0.30231 (6)	0.48259 (8)	0.0210 (3)	
N6	−0.24832 (18)	0.10919 (6)	0.64032 (8)	0.0253 (3)	
N9	0.47932 (15)	0.33680 (6)	0.49331 (7)	0.0194 (3)	
H9	0.5429	0.3194	0.5208	0.023*	
C40	0.64956 (18)	0.41244 (7)	0.51379 (8)	0.0189 (3)	
N7	−0.14356 (16)	0.20756 (6)	0.60938 (7)	0.0211 (3)	
H7	−0.2159	0.1904	0.5868	0.025*	
C39	0.50887 (18)	0.38841 (7)	0.47694 (9)	0.0195 (3)	
C28	−0.08427 (18)	0.18567 (7)	0.66985 (8)	0.0172 (3)	
C27	−0.15311 (18)	0.13184 (7)	0.68976 (8)	0.0178 (3)	
C36	−0.02993 (18)	0.32728 (7)	0.42846 (8)	0.0177 (3)	
C41	0.6897 (2)	0.46544 (8)	0.49946 (10)	0.0251 (4)	
H41	0.6331	0.4858	0.4661	0.030*	
C32	−0.26386 (18)	0.33195 (7)	0.35478 (9)	0.0195 (3)	
H32	−0.3619	0.3187	0.3437	0.023*	
C37	0.19986 (18)	0.32603 (7)	0.50953 (9)	0.0194 (3)	
H37A	0.2156	0.3153	0.5597	0.023*	
H37B	0.1901	0.3650	0.5085	0.023*	
C23	−0.3065 (2)	0.06086 (8)	0.65665 (10)	0.0282 (4)	
H23	−0.3722	0.0445	0.6230	0.034*	
C26	−0.11910 (18)	0.10871 (7)	0.75624 (9)	0.0187 (3)	
H26	−0.0554	0.1263	0.7896	0.022*	
C31	−0.17940 (18)	0.30923 (7)	0.41132 (8)	0.0175 (3)	
H31	−0.2227	0.2815	0.4384	0.021*	
C24	−0.2747 (2)	0.03374 (7)	0.72098 (10)	0.0256 (4)	
H24	−0.3149	−0.0004	0.7293	0.031*	
C30	0.01142 (18)	0.25116 (7)	0.51395 (9)	0.0186 (3)	
H30A	0.1032	0.2313	0.5285	0.022*	
H30B	−0.0431	0.2301	0.4775	0.022*	
C33	−0.20453 (19)	0.37409 (8)	0.31463 (9)	0.0238 (4)	
H33	−0.2608	0.3889	0.2762	0.029*	
C34	−0.0595 (2)	0.39369 (8)	0.33289 (10)	0.0262 (4)	
H34	−0.0198	0.4228	0.3073	0.031*	
C43	0.8975 (2)	0.45549 (8)	0.58395 (10)	0.0259 (4)	
H43	0.9832	0.4691	0.6088	0.031*	
C44	0.8496 (2)	0.40288 (7)	0.59447 (9)	0.0241 (4)	
H44	0.9063	0.3814	0.6265	0.029*	
C29	−0.0926 (2)	0.25855 (7)	0.57982 (9)	0.0210 (4)	
H29A	−0.0365	0.2783	0.6171	0.025*	
H29B	−0.1823	0.2797	0.5658	0.025*	
C42	0.8154 (2)	0.48745 (8)	0.53567 (10)	0.0272 (4)	
H42	0.8442	0.5230	0.5277	0.033*	
C38	0.34208 (19)	0.30960 (7)	0.46552 (9)	0.0211 (4)	
H38A	0.3565	0.2709	0.4686	0.025*	
H38B	0.3260	0.3190	0.4149	0.025*	
C35	0.02825 (19)	0.37092 (7)	0.38850 (9)	0.0218 (4)	
H35	0.1259	0.3846	0.3994	0.026*	
C25	−0.18174 (19)	0.05870 (7)	0.77240 (9)	0.0218 (4)	
H25	−0.1616	0.0423	0.8169	0.026*	
O1	0.51720 (13)	0.20741 (5)	0.55318 (6)	0.0224 (3)	
O2	0.92905 (13)	0.41536 (5)	0.82007 (6)	0.0233 (3)	
N5	1.22283 (16)	0.37938 (6)	0.69577 (7)	0.0204 (3)	
N3	0.55536 (15)	0.30360 (6)	0.77265 (7)	0.0206 (3)	
N2	0.35218 (16)	0.20593 (6)	0.64857 (7)	0.0210 (3)	
H2	0.2804	0.1881	0.6699	0.025*	
N4	0.97851 (15)	0.33578 (6)	0.76493 (7)	0.0183 (3)	
H4	1.0416	0.3178	0.7385	0.022*	
N1	0.24906 (17)	0.10774 (6)	0.61151 (8)	0.0251 (3)	
C5	0.35355 (18)	0.13041 (7)	0.56756 (9)	0.0183 (3)	
C6	0.41614 (18)	0.18486 (7)	0.58916 (8)	0.0175 (3)	
C17	1.00987 (18)	0.38738 (7)	0.78022 (8)	0.0179 (3)	
C18	1.15194 (18)	0.41036 (7)	0.74477 (9)	0.0179 (3)	
C9	0.47424 (18)	0.32890 (7)	0.82781 (8)	0.0174 (3)	
C11	0.24738 (19)	0.33473 (7)	0.90377 (9)	0.0201 (3)	
H11	0.1500	0.3219	0.9160	0.024*	
C22	1.3487 (2)	0.39964 (7)	0.66449 (9)	0.0225 (4)	
H22	1.4002	0.3784	0.6308	0.027*	
C21	1.4069 (2)	0.45028 (8)	0.67933 (10)	0.0259 (4)	
H21	1.4951	0.4628	0.6562	0.031*	
C14	0.53710 (19)	0.37270 (7)	0.86626 (9)	0.0223 (4)	
H14	0.6336	0.3863	0.8538	0.027*	
C15	0.69885 (18)	0.32599 (7)	0.74607 (9)	0.0190 (3)	
H15A	0.6910	0.3650	0.7458	0.023*	
H15B	0.7138	0.3142	0.6964	0.023*	
C8	0.50815 (18)	0.25151 (7)	0.74369 (9)	0.0188 (3)	
H8A	0.4570	0.2311	0.7814	0.023*	
H8B	0.5992	0.2316	0.7291	0.023*	
C13	0.4555 (2)	0.39580 (8)	0.92285 (10)	0.0258 (4)	
H13	0.4988	0.4247	0.9480	0.031*	
C10	0.32600 (18)	0.31133 (7)	0.84680 (8)	0.0176 (3)	
H10	0.2797	0.2835	0.8207	0.021*	
C12	0.3113 (2)	0.37699 (7)	0.94294 (9)	0.0241 (4)	
H12	0.2588	0.3923	0.9817	0.029*	
C7	0.39857 (19)	0.25749 (7)	0.67850 (9)	0.0208 (3)	
H7A	0.3071	0.2771	0.6933	0.025*	
H7B	0.4493	0.2784	0.6411	0.025*	
C16	0.84045 (19)	0.30946 (7)	0.79192 (9)	0.0198 (3)	
H16A	0.8245	0.3195	0.8422	0.024*	
H16B	0.8536	0.2707	0.7898	0.024*	
C1	0.1974 (2)	0.05894 (8)	0.59321 (10)	0.0290 (4)	
H1	0.1244	0.0427	0.6229	0.035*	
C4	0.40508 (19)	0.10617 (7)	0.50462 (9)	0.0219 (4)	
H4A	0.4750	0.1237	0.4747	0.026*	
C3	0.3509 (2)	0.05546 (8)	0.48688 (10)	0.0269 (4)	
H3	0.3842	0.0381	0.4451	0.032*	
C19	1.2014 (2)	0.46160 (8)	0.76317 (10)	0.0272 (4)	
H19	1.1488	0.4819	0.7975	0.033*	
C20	1.3310 (2)	0.48193 (8)	0.72935 (11)	0.0310 (4)	
H20	1.3666	0.5164	0.7401	0.037*	
C2	0.2463 (2)	0.03102 (7)	0.53257 (10)	0.0285 (4)	
H2A	0.2095	−0.0035	0.5228	0.034*	

### Atomic displacement parameters (Å^2^)

**Table:**

	*U*^11^	*U*^22^	*U*^33^	*U*^12^	*U*^13^	*U*^23^
O3	0.0171 (6)	0.0243 (6)	0.0225 (6)	−0.0026 (5)	−0.0031 (5)	−0.0003 (5)
O4	0.0194 (6)	0.0311 (7)	0.0280 (6)	0.0025 (5)	−0.0047 (5)	0.0053 (5)
N10	0.0197 (7)	0.0244 (8)	0.0191 (7)	−0.0006 (6)	−0.0016 (5)	−0.0002 (6)
N8	0.0154 (7)	0.0250 (8)	0.0226 (7)	−0.0057 (6)	−0.0056 (5)	0.0064 (6)
N6	0.0304 (8)	0.0253 (8)	0.0200 (7)	−0.0056 (6)	−0.0018 (6)	−0.0029 (6)
N9	0.0112 (7)	0.0268 (8)	0.0203 (7)	0.0014 (5)	−0.0017 (5)	0.0021 (6)
C40	0.0159 (8)	0.0244 (9)	0.0164 (8)	0.0026 (7)	0.0040 (6)	0.0003 (7)
N7	0.0205 (7)	0.0231 (8)	0.0196 (7)	−0.0061 (6)	−0.0039 (5)	0.0019 (6)
C39	0.0156 (8)	0.0264 (9)	0.0166 (8)	0.0031 (7)	0.0022 (6)	−0.0003 (7)
C28	0.0147 (8)	0.0208 (8)	0.0163 (8)	0.0016 (6)	0.0031 (6)	−0.0018 (6)
C27	0.0156 (8)	0.0195 (9)	0.0184 (8)	0.0007 (6)	0.0020 (6)	−0.0029 (6)
C36	0.0148 (8)	0.0224 (9)	0.0157 (8)	0.0014 (6)	0.0001 (6)	−0.0013 (6)
C41	0.0189 (9)	0.0280 (10)	0.0284 (9)	0.0027 (7)	0.0011 (7)	0.0054 (7)
C32	0.0130 (8)	0.0256 (9)	0.0198 (8)	0.0029 (6)	−0.0012 (6)	−0.0037 (7)
C37	0.0155 (8)	0.0240 (9)	0.0187 (8)	−0.0035 (6)	−0.0024 (6)	0.0016 (7)
C23	0.0348 (10)	0.0245 (10)	0.0253 (9)	−0.0089 (8)	−0.0011 (8)	−0.0053 (7)
C26	0.0134 (8)	0.0222 (9)	0.0205 (8)	0.0020 (6)	0.0013 (6)	−0.0013 (7)
C31	0.0155 (8)	0.0202 (8)	0.0170 (8)	0.0003 (6)	0.0023 (6)	−0.0020 (6)
C24	0.0270 (9)	0.0190 (9)	0.0310 (10)	−0.0018 (7)	0.0059 (7)	−0.0005 (7)
C30	0.0149 (8)	0.0206 (9)	0.0201 (8)	−0.0001 (6)	−0.0036 (6)	0.0021 (7)
C33	0.0189 (9)	0.0315 (10)	0.0209 (8)	0.0065 (7)	−0.0015 (7)	0.0043 (7)
C34	0.0233 (9)	0.0285 (10)	0.0269 (9)	0.0007 (7)	0.0022 (7)	0.0093 (8)
C43	0.0217 (9)	0.0307 (10)	0.0254 (9)	−0.0055 (7)	−0.0003 (7)	−0.0031 (8)
C44	0.0219 (9)	0.0292 (10)	0.0209 (8)	−0.0017 (7)	−0.0047 (7)	0.0017 (7)
C29	0.0244 (9)	0.0206 (9)	0.0181 (8)	−0.0013 (7)	−0.0006 (7)	0.0012 (7)
C42	0.0222 (9)	0.0243 (10)	0.0353 (10)	−0.0037 (7)	0.0042 (7)	0.0014 (8)
C38	0.0188 (8)	0.0254 (9)	0.0191 (8)	−0.0002 (7)	−0.0018 (6)	−0.0010 (7)
C35	0.0145 (8)	0.0266 (9)	0.0243 (9)	−0.0023 (7)	0.0000 (6)	0.0028 (7)
C25	0.0198 (8)	0.0220 (9)	0.0238 (9)	0.0040 (7)	0.0029 (7)	0.0037 (7)
O1	0.0169 (6)	0.0250 (6)	0.0253 (6)	−0.0022 (5)	0.0045 (5)	−0.0001 (5)
O2	0.0182 (6)	0.0255 (7)	0.0262 (6)	0.0028 (5)	0.0042 (5)	−0.0038 (5)
N5	0.0199 (7)	0.0221 (8)	0.0192 (7)	−0.0011 (6)	0.0012 (5)	−0.0008 (6)
N3	0.0152 (7)	0.0252 (8)	0.0214 (7)	−0.0044 (6)	0.0057 (5)	−0.0062 (6)
N2	0.0204 (7)	0.0239 (8)	0.0187 (7)	−0.0054 (6)	0.0038 (5)	−0.0026 (6)
N4	0.0126 (6)	0.0212 (7)	0.0211 (7)	0.0018 (5)	0.0015 (5)	−0.0030 (6)
N1	0.0278 (8)	0.0275 (8)	0.0199 (7)	−0.0065 (6)	0.0022 (6)	0.0007 (6)
C5	0.0146 (8)	0.0220 (9)	0.0183 (8)	0.0008 (6)	−0.0011 (6)	0.0026 (7)
C6	0.0131 (8)	0.0218 (9)	0.0177 (8)	0.0015 (6)	−0.0027 (6)	0.0004 (6)
C17	0.0146 (8)	0.0226 (9)	0.0165 (8)	0.0032 (6)	−0.0032 (6)	0.0004 (7)
C18	0.0159 (8)	0.0210 (9)	0.0168 (8)	0.0026 (6)	−0.0033 (6)	0.0010 (6)
C9	0.0145 (8)	0.0207 (9)	0.0172 (8)	0.0026 (6)	0.0003 (6)	0.0015 (6)
C11	0.0156 (8)	0.0251 (9)	0.0197 (8)	0.0050 (7)	0.0016 (6)	0.0042 (7)
C22	0.0214 (9)	0.0273 (9)	0.0188 (8)	−0.0027 (7)	0.0035 (6)	−0.0019 (7)
C21	0.0233 (9)	0.0286 (10)	0.0260 (9)	−0.0062 (7)	0.0043 (7)	0.0012 (7)
C14	0.0160 (8)	0.0250 (9)	0.0257 (9)	−0.0005 (7)	0.0009 (7)	−0.0039 (7)
C15	0.0146 (8)	0.0240 (9)	0.0185 (8)	−0.0029 (6)	0.0033 (6)	−0.0022 (7)
C8	0.0161 (8)	0.0215 (9)	0.0190 (8)	0.0001 (6)	0.0037 (6)	−0.0029 (7)
C13	0.0199 (9)	0.0261 (10)	0.0312 (10)	0.0028 (7)	−0.0015 (7)	−0.0093 (8)
C10	0.0164 (8)	0.0198 (8)	0.0166 (8)	0.0007 (6)	−0.0013 (6)	0.0021 (6)
C12	0.0231 (9)	0.0275 (9)	0.0216 (8)	0.0081 (7)	0.0021 (7)	−0.0040 (7)
C7	0.0203 (8)	0.0216 (9)	0.0205 (8)	−0.0005 (7)	0.0011 (6)	−0.0017 (7)
C16	0.0182 (8)	0.0214 (9)	0.0198 (8)	0.0001 (7)	0.0033 (6)	0.0009 (7)
C1	0.0351 (10)	0.0271 (10)	0.0250 (9)	−0.0109 (8)	0.0020 (8)	0.0043 (8)
C4	0.0189 (8)	0.0243 (9)	0.0225 (8)	0.0006 (7)	0.0020 (6)	−0.0002 (7)
C3	0.0299 (10)	0.0250 (10)	0.0260 (9)	0.0030 (7)	0.0010 (7)	−0.0057 (7)
C19	0.0217 (9)	0.0260 (10)	0.0339 (10)	−0.0010 (7)	0.0048 (7)	−0.0086 (8)
C20	0.0259 (10)	0.0246 (10)	0.0427 (11)	−0.0076 (8)	0.0060 (8)	−0.0068 (8)
C2	0.0345 (10)	0.0199 (9)	0.0310 (10)	−0.0042 (8)	−0.0046 (8)	0.0006 (8)

### Geometric parameters (Å, º)

**Table:**

O3—C28	1.236 (2)	O1—C6	1.235 (2)
O4—C39	1.227 (2)	O2—C17	1.232 (2)
N10—C40	1.340 (2)	N5—C18	1.338 (2)
N10—C44	1.337 (2)	N5—C22	1.335 (2)
N8—C36	1.390 (2)	N3—C9	1.390 (2)
N8—C37	1.449 (2)	N3—C15	1.449 (2)
N8—C30	1.452 (2)	N3—C8	1.455 (2)
N6—C27	1.344 (2)	N2—H2	0.8600
N6—C23	1.336 (2)	N2—C6	1.337 (2)
N9—H9	0.8600	N2—C7	1.448 (2)
N9—C39	1.341 (2)	N4—H4	0.8600
N9—C38	1.454 (2)	N4—C17	1.338 (2)
C40—C39	1.509 (2)	N4—C16	1.452 (2)
C40—C41	1.387 (3)	N1—C5	1.342 (2)
N7—H7	0.8600	N1—C1	1.333 (2)
N7—C28	1.336 (2)	C5—C6	1.508 (2)
N7—C29	1.448 (2)	C5—C4	1.383 (2)
C28—C27	1.509 (2)	C17—C18	1.508 (2)
C27—C26	1.381 (2)	C18—C19	1.383 (3)
C36—C31	1.400 (2)	C9—C14	1.404 (2)
C36—C35	1.405 (2)	C9—C10	1.401 (2)
C41—H41	0.9300	C11—H11	0.9300
C41—C42	1.382 (3)	C11—C10	1.383 (2)
C32—H32	0.9300	C11—C12	1.385 (3)
C32—C31	1.385 (2)	C22—H22	0.9300
C32—C33	1.382 (3)	C22—C21	1.380 (3)
C37—H37A	0.9700	C21—H21	0.9300
C37—H37B	0.9700	C21—C20	1.381 (3)
C37—C38	1.534 (2)	C14—H14	0.9300
C23—H23	0.9300	C14—C13	1.388 (2)
C23—C24	1.387 (3)	C15—H15A	0.9700
C26—H26	0.9300	C15—H15B	0.9700
C26—C25	1.388 (2)	C15—C16	1.536 (2)
C31—H31	0.9300	C8—H8A	0.9700
C24—H24	0.9300	C8—H8B	0.9700
C24—C25	1.382 (3)	C8—C7	1.529 (2)
C30—H30A	0.9700	C13—H13	0.9300
C30—H30B	0.9700	C13—C12	1.384 (3)
C30—C29	1.528 (2)	C10—H10	0.9300
C33—H33	0.9300	C12—H12	0.9300
C33—C34	1.383 (3)	C7—H7A	0.9700
C34—H34	0.9300	C7—H7B	0.9700
C34—C35	1.388 (3)	C16—H16A	0.9700
C43—H43	0.9300	C16—H16B	0.9700
C43—C44	1.384 (3)	C1—H1	0.9300
C43—C42	1.382 (3)	C1—C2	1.384 (3)
C44—H44	0.9300	C4—H4A	0.9300
C29—H29A	0.9700	C4—C3	1.381 (3)
C29—H29B	0.9700	C3—H3	0.9300
C42—H42	0.9300	C3—C2	1.381 (3)
C38—H38A	0.9700	C19—H19	0.9300
C38—H38B	0.9700	C19—C20	1.382 (3)
C35—H35	0.9300	C20—H20	0.9300
C25—H25	0.9300	C2—H2A	0.9300
			
C44—N10—C40	116.80 (15)	C22—N5—C18	117.06 (15)
C36—N8—C37	121.20 (14)	C9—N3—C15	120.88 (14)
C36—N8—C30	121.79 (13)	C9—N3—C8	121.83 (13)
C37—N8—C30	117.01 (13)	C15—N3—C8	117.07 (13)
C23—N6—C27	116.90 (15)	C6—N2—H2	118.6
C39—N9—H9	119.3	C6—N2—C7	122.80 (14)
C39—N9—C38	121.41 (14)	C7—N2—H2	118.6
C38—N9—H9	119.3	C17—N4—H4	119.2
N10—C40—C39	117.07 (15)	C17—N4—C16	121.59 (13)
N10—C40—C41	123.47 (16)	C16—N4—H4	119.2
C41—C40—C39	119.42 (15)	C1—N1—C5	117.02 (15)
C28—N7—H7	118.2	N1—C5—C6	117.30 (14)
C28—N7—C29	123.57 (14)	N1—C5—C4	123.14 (16)
C29—N7—H7	118.2	C4—C5—C6	119.55 (14)
O4—C39—N9	123.26 (16)	O1—C6—N2	124.18 (16)
O4—C39—C40	121.20 (16)	O1—C6—C5	121.22 (14)
N9—C39—C40	115.52 (14)	N2—C6—C5	114.60 (14)
O3—C28—N7	124.11 (15)	O2—C17—N4	123.34 (15)
O3—C28—C27	121.46 (14)	O2—C17—C18	120.88 (15)
N7—C28—C27	114.42 (14)	N4—C17—C18	115.78 (14)
N6—C27—C28	116.48 (14)	N5—C18—C17	117.03 (15)
N6—C27—C26	123.36 (15)	N5—C18—C19	123.43 (15)
C26—C27—C28	120.16 (15)	C19—C18—C17	119.54 (15)
N8—C36—C31	120.78 (15)	N3—C9—C14	121.52 (14)
N8—C36—C35	121.65 (15)	N3—C9—C10	120.73 (15)
C31—C36—C35	117.57 (15)	C10—C9—C14	117.75 (15)
C40—C41—H41	120.7	C10—C11—H11	119.5
C42—C41—C40	118.70 (17)	C10—C11—C12	121.09 (16)
C42—C41—H41	120.7	C12—C11—H11	119.5
C31—C32—H32	119.5	N5—C22—H22	118.1
C33—C32—H32	119.5	N5—C22—C21	123.70 (16)
C33—C32—C31	120.95 (15)	C21—C22—H22	118.1
N8—C37—H37A	108.9	C22—C21—H21	120.8
N8—C37—H37B	108.9	C22—C21—C20	118.38 (16)
N8—C37—C38	113.23 (14)	C20—C21—H21	120.8
H37A—C37—H37B	107.7	C9—C14—H14	120.0
C38—C37—H37A	108.9	C13—C14—C9	120.08 (16)
C38—C37—H37B	108.9	C13—C14—H14	120.0
N6—C23—H23	118.1	N3—C15—H15A	109.0
N6—C23—C24	123.76 (17)	N3—C15—H15B	109.0
C24—C23—H23	118.1	N3—C15—C16	113.09 (14)
C27—C26—H26	120.6	H15A—C15—H15B	107.8
C27—C26—C25	118.81 (16)	C16—C15—H15A	109.0
C25—C26—H26	120.6	C16—C15—H15B	109.0
C36—C31—H31	119.4	N3—C8—H8A	109.3
C32—C31—C36	121.14 (15)	N3—C8—H8B	109.3
C32—C31—H31	119.4	N3—C8—C7	111.76 (14)
C23—C24—H24	120.8	H8A—C8—H8B	107.9
C25—C24—C23	118.43 (17)	C7—C8—H8A	109.3
C25—C24—H24	120.8	C7—C8—H8B	109.3
N8—C30—H30A	109.2	C14—C13—H13	119.1
N8—C30—H30B	109.2	C12—C13—C14	121.79 (17)
N8—C30—C29	112.07 (14)	C12—C13—H13	119.1
H30A—C30—H30B	107.9	C9—C10—H10	119.5
C29—C30—H30A	109.2	C11—C10—C9	121.06 (16)
C29—C30—H30B	109.2	C11—C10—H10	119.5
C32—C33—H33	120.8	C11—C12—H12	120.9
C32—C33—C34	118.44 (16)	C13—C12—C11	118.16 (15)
C34—C33—H33	120.8	C13—C12—H12	120.9
C33—C34—H34	119.2	N2—C7—C8	112.33 (14)
C33—C34—C35	121.54 (16)	N2—C7—H7A	109.1
C35—C34—H34	119.2	N2—C7—H7B	109.1
C44—C43—H43	120.7	C8—C7—H7A	109.1
C42—C43—H43	120.7	C8—C7—H7B	109.1
C42—C43—C44	118.59 (17)	H7A—C7—H7B	107.9
N10—C44—C43	123.74 (17)	N4—C16—C15	110.18 (13)
N10—C44—H44	118.1	N4—C16—H16A	109.6
C43—C44—H44	118.1	N4—C16—H16B	109.6
N7—C29—C30	112.14 (14)	C15—C16—H16A	109.6
N7—C29—H29A	109.2	C15—C16—H16B	109.6
N7—C29—H29B	109.2	H16A—C16—H16B	108.1
C30—C29—H29A	109.2	N1—C1—H1	118.2
C30—C29—H29B	109.2	N1—C1—C2	123.69 (17)
H29A—C29—H29B	107.9	C2—C1—H1	118.2
C41—C42—C43	118.68 (17)	C5—C4—H4A	120.5
C41—C42—H42	120.7	C3—C4—C5	118.91 (16)
C43—C42—H42	120.7	C3—C4—H4A	120.5
N9—C38—C37	110.24 (13)	C4—C3—H3	120.7
N9—C38—H38A	109.6	C4—C3—C2	118.60 (17)
N9—C38—H38B	109.6	C2—C3—H3	120.7
C37—C38—H38A	109.6	C18—C19—H19	120.8
C37—C38—H38B	109.6	C20—C19—C18	118.40 (17)
H38A—C38—H38B	108.1	C20—C19—H19	120.8
C36—C35—H35	119.9	C21—C20—C19	119.02 (18)
C34—C35—C36	120.28 (16)	C21—C20—H20	120.5
C34—C35—H35	119.9	C19—C20—H20	120.5
C26—C25—H25	120.7	C1—C2—H2A	120.7
C24—C25—C26	118.65 (16)	C3—C2—C1	118.58 (17)
C24—C25—H25	120.7	C3—C2—H2A	120.7

### Hydrogen-bond geometry (Å, º)

Cg5 and Cg6 are the centroids of the C9–C14 benzene and C31–C36 benzene rings, respectively.

**Table:**

*D*—H···*A*	*D*—H	H···*A*	*D*···*A*	*D*—H···*A*
N2—H2···O3	0.86	2.44	3.1099 (18)	135
N7—H7···O1^i^	0.86	2.42	3.0998 (18)	136
C24—H24···O2^ii^	0.93	2.48	3.311 (2)	149
C25—H25···O4^iii^	0.93	2.54	3.213 (2)	129
C8—H8*A*···*Cg*6^iv^	0.97	2.72	3.6468 (18)	161
C30—H30*B*···*Cg*5^v^	0.97	2.74	3.6584 (18)	159

Symmetry codes: (i) *x*−1, *y*, *z*; (ii) −*x*+1/2, *y*−1/2, −*z*+3/2; (iii) *x*−1/2, −*y*+1/2, *z*+1/2; (iv) *x*+1/2, −*y*+1/2, *z*+1/2; (v) *x*−1/2, −*y*+1/2, *z*−1/2.
